# Apoptosis in the Mammary Gland of Virgin Rats Subchronically Fed With a Vitamin A Deficient Diet

**DOI:** 10.1155/omcl/6334165

**Published:** 2025-07-14

**Authors:** M. Vasquez Gomez, V. Filippa, M. Acosta, F. Mohamed, F. Campo Verde, C. Ferrari, G. A. Jahn, M. S. Giménez, D. C. Ramirez, S. E. Gomez Mejiba

**Affiliations:** ^1^Laboratory of Nutrition, Environment, and Metabolism, CONICET-San Luis, National University of San Luis, San Luis, Argentina; ^2^Laboratory of Histology, National University of San Luis, San Luis, Argentina; ^3^Laboratory of Reproduction and Breastfeeding, CONICET-Mendoza, Mendoza, Argentina; ^4^Laboratory of Experimental and Translational Medicine, CONICET-San Luis, National University of San Luis, Argentina; ^5^Laboratory of Nutrition and Experimental Therapeutics, CONICET-San Luis, National University of San Luis, San Luis, Argentina

**Keywords:** apoptosis, inflammation, mammary gland dysfunction, subchronic, vitamin A deficiency

## Abstract

Mammary gland epithelial dysfunction is one of the serious consequences of subchronic dietary vitamin A deficiency (VAD). However, the underlying mechanism of this process is incompletely known. Consequently, we utilized a virgin rat model of dietary VAD (3 and 6 months) and subsequently intervened with a vitamin A sufficient (VAS) diet (0.5 or 1 month) prior to treatment completion. This experimental model allowed us to investigate the underlying molecular mechanism of mammary gland tissue dysfunction caused by VAD. Dietary VAD for 3 and 6 months caused increased inflammatory cell infiltration in the mammary gland parenchyma and glandular cells, with increased inflammation and apoptosis and reduced cell proliferation. These changes can be reversed with a VAS diet. Imbalances between the NF-κB and retinoic acid (RA) signaling pathways underlie mammary gland dysfunction following subchronic VAD. Nulliparous rats fed a VAD diet experience mammary gland epithelial dysfunction because of inflammation, apoptosis, and impaired cell growth.

## 1. Introduction

Worldwide, vitamin A deficiency (VAD) affects an estimated 190 million preschool-aged children and 19.1 million pregnant women in developing countries [1–4], and it increases morbidity and mortality due to increased susceptibility to infection [5]. Vitamin A and its derivatives (referred to as retinoids) are essential dietary compounds and are key regulators of cell differentiation, proliferation, inflammation, and death [3, 6]. See [3, 7] for the most recent and compressive reviews on the topic. Adult animals deprived of dietary vitamin A display severe abnormalities, including dysfunction of the epithelia of the mammary gland, the mechanism of which is only partially known [8].

The mammary gland is an interesting tissue for the study of apoptosis and its related signaling factors and underlying mechanisms [9]. In fact, mammary gland development is characterized by stages of proliferation during puberty, pregnancy, and lactation, followed by an involution phase, when extensive tissue restructuring, involving the removal of a proportion of secretory epithelial cells, occurs [10]. In the cell death program, the involution phase is one of the most dramatic physiological responses, in which approximately 80% of mammary epithelial cells undergo programed cell death [11]. One of the most important regulators of apoptosis via the mitochondrial pathway is the BCL2 protein family [9, 12]. The proapoptotic proteins BAX, BAK, and BAD, as well as the death suppressor proteins BCL-X, BCL2, and BCL-W, are synthesized in the mammary gland [13]. Dynamic changes in the expression profiles of these proteins occur during development, suggesting that these changes may be involved in the regulation of postlactation apoptosis [14]. In vivo, cell survival programs can be determined by extracellular signals that directly affect the levels and functions of members of the BCL2 family [12].

In mouse mammary gland tissue, BCL2 gene expression is undetectable during involution, whereas the BAX protein is detected in many cells, and its expression increases during the first stage of involution [15]. Similarly, BAX mRNA levels increase with the onset of involution [16]. On the other hand, in the mammary glands of BAX^−/−^ rats, involution is delayed at first but resembles that in the wild-type gland at 10 days after involution, when remodeling is complete [17]. In a WAP-BAX transgenic mouse that expresses the gene encoding BAX under the transcriptional control of the whey acidic protein (WAP) promoter, a model of forced mammary gland involution, the positive regulation of a single proapoptotic factor of the BCL2 protein family was demonstrated to be sufficient to initiate the apoptosis of functionally differentiated mammary epithelial cells [18]. However, it is unknown whether VAD causes apoptosis in the mammary glands of virgin rats.

Retinoic acid (RA) interacts with nuclear receptors, such as the retinoic acid receptor (RAR) and retinoid X receptor (RXR), to regulate the transcription of several target genes by binding to retinoic acid-responsive elements (RAREs) in their regulatory regions [19]. These receptors form heterodimers; RAR comprises three major isoforms (α, β, and γ) that interact with all forms of RA, whereas RXR, which also has the α, β, and γ isoforms, mainly interacts with *9-cis* RA. RA and its interaction with its cognate receptors RARα or RXR are critical for appropriate mammary gland differentiation and morphogenesis [3, 20]. The binding of RA-RARα/RXR to RAREs controls the expression of a number of genes involved in cell differentiation, growth, proliferation, and immunity [21].

The NF-κB signaling pathway has important functions in cellular interactions, cell survival, and differentiation [22]. These functions are mediated by the expression of several proinflammatory cytokines (e.g., TNFα and IL-6), enzymes (e.g., inducible nitric oxide synthase [iNOS] and cyclooxygenase-2 [COX-2]), chemokines (e.g., CCL2 and CXCL10), adhesion molecules (e.g., ICAM-1 and VCAM-1) and proapoptotic genes, such as BAX [23]. Vitamin A has an anti-inflammatory effect due to its binding to RAREs, which are consensus cis elements found in several anti-inflammatory gene regulatory parts [24]. Therefore, it is possible that, under a VAD diet, the tight balance between NF-κB and RA-activated RAR/RXR signaling is altered, in favor of NF-κB signaling, in the mammary gland, causing tissue structural changes linked to apoptosis, proliferation, and cell differentiation.

Herein, we investigated whether VAD causes apoptosis in the mammary glands of nulliparous rats and investigated the underlying mechanisms by which this process occurs.

A preprint has previously been published [25].

## 2. Materials and Methods

### 2.1. Experimental Model

Female Wistar rats bred in our animal facilities (IMIBIO-SL, National University of San Luis, Argentina) were used. The rats were weaned at 21 days of age and immediately randomly assigned to 6 experimental groups. The experimental groups were as follows (Supporting Information 1: Figure S1 for a scheme of the experimental design): c3m, rats fed a vitamin A sufficient (VAS) diet for 3 months; d3m, rats fed a VAD diet for 3 months; r3m, rats fed a VAD diet for 2.5 months followed by a VAS diet for 0.5 months; c6m, rats fed a VAS diet; d6m, rats fed a VAD diet for 6 months; and r6m, rats fed a VAD diet for 5 months followed by a VAS diet for 1 month. Diets were prepared according to AIN-93G for laboratory rodents [26]. The compositions of the VAD and VAS diets are shown in Supporting Information 2: Table S1, and the compositions of the mineral and vitamin mixtures are shown in Supporting Information 2: Tables S2 and S3, respectively. The only difference between the VAD and VAS diets is that the vitamin mixture added to the VAD does not contain trans-retinyl palmitate. The rats were housed in individual cages and kept in a 21–23°C controlled environment with a 12-h light:dark cycle. They were given free access to food and water throughout the entire 3- and 6-month period of diet. After the entire treatment period, the rats were euthanized by CO_2_ inhalation. All the experiments with animals were performed according to the National Institutes of Health Guide for the Care and Use of Laboratory Animals [27] and the National University of San Luis Committee's Guidelines for the Care and Use of Experimental Animals (approved UNSL-CICUAL-Protocol# B-202-3/15-FQByF-RD-324-16).

### 2.2. Measurement of Retinol Concentrations in the Plasma And Mammary Glands

The blood samples were collected in EDTA-treated tubes. To minimize photoisomerization of vitamin A, the plasma was taken under reduced yellow light and frozen in the dark at −70°C until the retinol concentration was determined. Analyses were carried out within 1–3 weeks of sample collection. The retinol concentration was determined using the modified technique of Neeld and Pearson [28]. Briefly, the plasma was treated with 1 ml of 95% ethanol to precipitate proteins. The supernatant was then treated with 1.5 ml of petroleum ether to extract vitamin A and carotenoids. After centrifugation at 1200 × *g* for 10 min, the absorbance of the supernatant at 450 nm, corresponding to the β-carotene absorbance, was read, and the sample was then dried in an oven at 37°C. The residue was removed in 50 µl of chloroform, 50 µl of acetic anhydride, and 500 µl of trifluoroacetic acid with vigorous stirring. The samples were read within 30 s at 620 nm. A standard curve of vitamin A and β-carotenes was generated. Because β-carotenes react with trifluoroacetic acid, the results were corrected after the absorbance at 450 nm was read and the corresponding correction factor was calculated.

### 2.3. Histology of the Mammary Gland

For the light microscopy studies, four mammary glands from each group were excised and fixed in Bouin's fluid. The samples were then dehydrated in an increasing ethanol series, cleared in xylene, and embedded in paraffin. Sections (5 μm thick) were obtained using a Reichert-Jung Hn 40 sliding microtome. In these sections, hematoxylin and eosin (H&E) staining, immunohistochemistry, and TUNEL assays were performed.

### 2.4. Immunohistochemistry

BCL2– and BAX–positive cells were detected by immunohistochemistry in paraffin slides of the mammary gland. Briefly, the sections were first deparaffinized with xylene, hydrated through decreasing concentrations of ethanol, and rinsed with distilled water and phosphate-buffered saline (PBS, 0.01 M, pH 7.4). Antigen retrieval was performed by microwaving the sections for 6 min (2 × 3 min) at 900 W in sodium citrate buffer (0.01 M, pH 6.0). Endogenous peroxidase activity was inhibited with 3% H_2_O_2_ in water for 20 min. Nonspecific binding sites for immunoglobulins were blocked by incubation for 20 min with normal mouse serum diluted in PBS containing 1% bovine serum albumin, 0.09% sodium azide, and 0.1% Tween-20. The sections were incubated with the following primary antibodies: 8 h in a humidified chamber at 4°C with mouse monoclonal antihuman BCL-2 (clon BCL-2/100; Catalog. No. AM287-5M, BioGenex, San Ramón, CA, USA), 30 min in a humidified chamber at 20°C with rabbit polyclonal antihuman BAX protein (Catalog. No. AR347-5R, BioGenex, San Ramón, CA, USA), and for 4 h in a humidified chamber at 20°C with mouse monoclonal anti-rat proliferating cell nuclear antigen (PCNA) (clon PC10; Catalog. No. AM252-5M, BioGenex, San Ramón, CA, USA). After rinsing with PBS for 10 min, immunohistochemical visualization was carried out using the Super Sensitive Ready-to-Use Immunostaining Kit (BioGenex, San Ramon, CA, USA), which was used as follows: the sections were incubated for 30 min with diluted biotinylated anti-IgG and, after being washed in PBS, were incubated for 30 min with horseradish peroxidase-conjugated streptavidin and finally washed in PBS. The reaction sites were visualized using a freshly prepared solution containing 100 µL of 3,3′-diaminobenzidine tetrahydrochloride chromogen in 2.5 mL of PBS and 50 µL of H_2_O_2_ substrate solution. The sections were counterstained with Harris' hematoxylin for 10 s, dehydrated, and mounted. For the negative control for immunohistochemistry, the following procedure was performed: 10% normal serum and PBS were used to replace the primary antibodies. No positive structures or cells were found in these sections. The large and small intestines of the rats were used as positive controls.

### 2.5. Tunel Assay

The terminal deoxynucleotidyl transferase (TdT)-mediated biotinylated dUTP nick-end labeling (TUNEL) assay (Dead-End Colorimetric TUNEL System, Promega, Madison, WI) was used according to the manufacturer's instructions. A positive control was run after pretreatment with DNAse I. A negative control was produced by using the equilibrium buffer mixture with biotinylated nucleotides but without the TdT enzyme. The slides were counterstained with hematoxylin and mounted without contrast agents.

### 2.6. Morphometric Analysis of Immunohistochemistry Images

The mammary gland slides used for assessing BCL2-, BAX-, TUNEL-, and PCNA-positive cells were examined using an Olympus BX-40 light microscope. Immunohistochemical images were captured with a Sony SSC-DC5OA camera and processed with Image-Pro Plus 5.0 software. Briefly, the image was displayed on a color monitor; a standard area of 18141.82 µm^2^ (reference area) was defined on the monitor, and distance calibration was performed using a slide with a micrometric scale for microscopy (Reichert, Austria). The morphometric study was performed as follows: three regularly spaced serial tissue sections (100 µm each) from mammary glands were used, and microscopic fields were examined under 400 × magnification. In each section, 10 microscopic fields were randomly selected throughout the mammary gland. In each image, the percentages of TUNEL- and PCNA-positive cells were determined utilizing the following formula: percentage of positive cells = positive cells/total cell count × 100.

BAX^+^ and BCL2^+^ cells were measured using the Image-Pro Plus 3.0.1 system (Media Cybernetics, Silver Spring, MA, USA). For the immunohistochemistry technique, images were digitized by a color charge-coupled device (CCD) video camera (Sony, Montvale, NJ, USA) mounted on a conventional light microscope (Olympus BH-2; Olympus Company, Tokyo, Japan) at a magnification of 400x. The microscope was prepared for Koehler illumination. This was achieved by recording a reference image of an empty field for the correction of unequal illumination (shading correction) and by calibrating the measurement system with a reference slide to determine background threshold values. The reference slides contained a series of tissue sections stained in the absence of a primary antibody. The positive controls were used as interassay controls to maximize the accuracy and robustness of the method. The methodological details of image analysis have been described previously [29]. Using a color segmentation analysis tool, the total intensity of the positively stained cytoplasmic area (brown reaction product) was measured and is shown as a ratio (%) of the total cytoplasmic area (brown reaction product blue hematoxylin). The image analysis score was calculated separately by using AutoPro macro language, an automated sequence operation created to measure the immunohistochemically stained area (IHCSA). The IHCSA was calculated as a percentage of the total area evaluated through color segmentation analysis, which extracts objects by locating all objects of a specific color (brown stain). The brown stain was selected with a sensitivity of 4 (maximum of 5), and a mask was then applied to separate the colors permanently. The images were then transformed to a bilevel scale tagged image file format (TIFF). The IHCSA (black area) was calculated from at least 50 images of each area of the mammary gland in each slide being studied.

### 2.7. RNA Isolation and RT–qPCR Analysis

The mammary tissues were homogenized with an Ultra-Turrax T-25 digital homogenizer. Total RNA was isolated from 150–200 mg of mammary tissue using the guanidinium isothiocyanate-acid phenol method as modified by Puissant and Houdebine [30]. Ten micrograms of total RNA were reverse transcribed (RT) at 37°C using random hexamer primers and Moloney murine leukemia virus retrotranscriptase (Invitrogen-Life Technologies, Buenos Aires, Argentina) in a 20 µL reaction mixture. The RNA was first denatured at 70°C for 5 min in the presence of 2.5 µg of random hexamer primers (Invitrogen). For the subsequent RT reaction, the following mixture was added: RT buffer (50 mM Tris-HCl [pH 8.4], 75 mM KCl, 3 mM MgCl_2_), 0.5 mM dNTPs, 5 mM DTT, and 200 units of M-MLV reverse transcriptase. The mixture was incubated at 37°C for 50 min. Next, the mixture was inactivated by heating at 70°C for 15 min. The cDNA was stored at −20°C. The mRNA levels of BAX, BCL-2, and S16 were estimated using real-time RT–PCR using the rat-specific primers and reaction conditions described in Supporting Information 2: Table S4. The PCRs were performed using a Corbett Rotor-Gene 6000 Real-Time Thermocycler (Corbett Research Pty Ltd. Sydney, Australia) and Eva-Green (Biotium Hayward, CA) in a final volume of 20 µL. The reaction mixture consisted of 2 µL of 10x PCR buffer, 1 µL of 50 mM MgCl_2_, 0.4 µL of 10 mM dNTP mixture (Invitrogen), 1 µL of Eva Green, 0.25 µL of 5 U/mL Taq DNA Polymerase (Invitrogen), 0.1 µL of each 2.5 mM primer (forward and reverse primers) and 10 µL of diluted cDNA. The PCRs were initiated by incubation for 5 min at 95°C, followed by 40 cycles. Melting curve analysis was used to verify that a single specific amplified product was generated. Real-time quantification was monitored by measuring the increase in fluorescence caused by the binding of EvaGreen dye to double-stranded DNA at the end of each amplification cycle. Relative expression was determined using the comparative quantitation method of normalized samples with the expression of a calibrator sample, according to the manufacturer's protocol [31]. Each PCR run included a no-template control and a sample without RT. All the measurements were performed in duplicate. The reaction conditions and quantities of cDNA added were calibrated so that the assay response was linear for the amount of input cDNA for each pair of primers. The RNA samples were assessed for DNA contamination by performing different PCRs without prior RT. Relative levels of mRNA were normalized to the S16 or S28 housekeeping gene expression. The real-time PCR products were analyzed on 2% agarose gels containing 0.5 mg/mL ethidium bromide, and a unique band of approximately correct molecular weight corresponded with a unique peak in the melt curve analysis.

### 2.8. Statistics

The data were normally distributed according to the Shapiro–Wilk test. The data are shown as representative images or mean values ± standard deviations (SD) and were analyzed by one-way ANOVA followed by Tukey's post hoc test for specific comparisons. *p*  < 0.05 was considered to indicate statistical significance. GraphPad Prism (v. 3.02) software was used for statistical analysis.

## 3. Results

### 3.1. Dietary VAD Affects Body Weight Gain in Virgin Rats

Body weight gains after 3 or 6 months of diet were determined in each experimental group (Table 1). After 3 or 6 months of diet, animals fed the VAD diet gained less weight than those fed the VAS diet did (c3m vs. d3m and c6m vs. d6m). These changes were reversed in the r3m and r6m groups, with a significant difference (*p*  < 0.05) between the r3m and d3m groups and the r6m and d6m groups.

### 3.2. Dietary VAD Affects the Histological Architecture of the Mammary Gland

**Figure 1:**
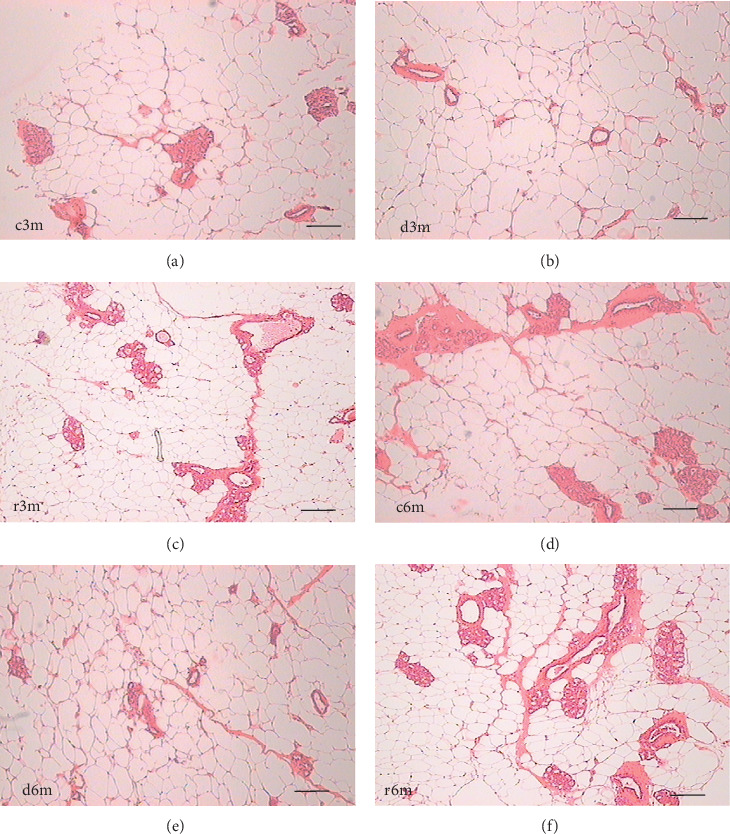
Mammary gland structural changes caused by VAD. H&E staining of mammary glands isolated from the experimental groups at (A) c3m, (B) d3m, (C) r3m, (D) c6m, (E) d6m, and (F) r6m. Magnification 40×. The scale bar is 100 µm.

To determine the effect of VAD on the tissue architecture of the mammary gland, we performed a morphological study on H&E-stained slides (Figure 1). In the mammary glands of the c3m rats, ductal development was observed; however, it was not observed in the d3m rats. In the mammary glands of the c3m group, there were more ducts than in those of the deficient group, and incipient alveolar lobe arborization had begun. In the r3m rats, modest alveolar lobe development was observed, which was greater than that in the d3m rats. In the d6m group, lower parenchymal development (i.e., fewer acini or alveoli) was observed, highlighting the ductal predominance compared with that in the c6m group. In the animals from the r6m group, the recovery of the size and number of mammary alveoli was notable, similar to that observed in the c6m group. Subchronic deficiency of dietary vitamin A causes mammary gland dysfunction in the mammary glands of nulliparous rats. These findings are consistent with the abnormal development of the mammary gland parenchyma. Dietary vitamin A supplementation prevents mammary gland dysfunction in nulliparous rats.

### 3.3. Dietary VAD Alters the RA Concentration in the Serum, Liver, and Mammary Gland

The effects of diet on the concentration of RA in the serum, liver, and mammary glands of the animals in each experimental group (Supporting Information 1: Figure S1) are shown in Table 2. A VAD diet for 3 months (d3m) caused a reduction in the RA concentration in the serum, liver, and mammary glands. However, refeeding animals for 15 days (r3m) only partially reestablished (one-third) the concentration of RA found in those tissues from animals fed a VAS diet for 3 months (c3m). The concentrations of RA in the serum, liver, and mammary glands of animals fed a VAS diet for 3 or 6 months were similar (c3m vs. c6m). However, the RA concentrations in the serum of the animals fed the VAD diet for 6 months were ~30 times lower than those in the same diet for 3 months (d6m vs. d3m). The effect of refeeding a VAS diet for 1 month increased the RA serum and liver concentrations, similar to those of feeding a VAS diet for 6 months (r6m vs. c6m). However, the concentration of RA in the mammary gland increased, but it was only approximately one-third of that found in the mammary gland of rats fed a VAS for 6 months (r6m vs. c6m).

### 3.4. Dietary VAD Causes Apoptosis in the Mammary Glands of Virgin Rats

**Figure 2:**
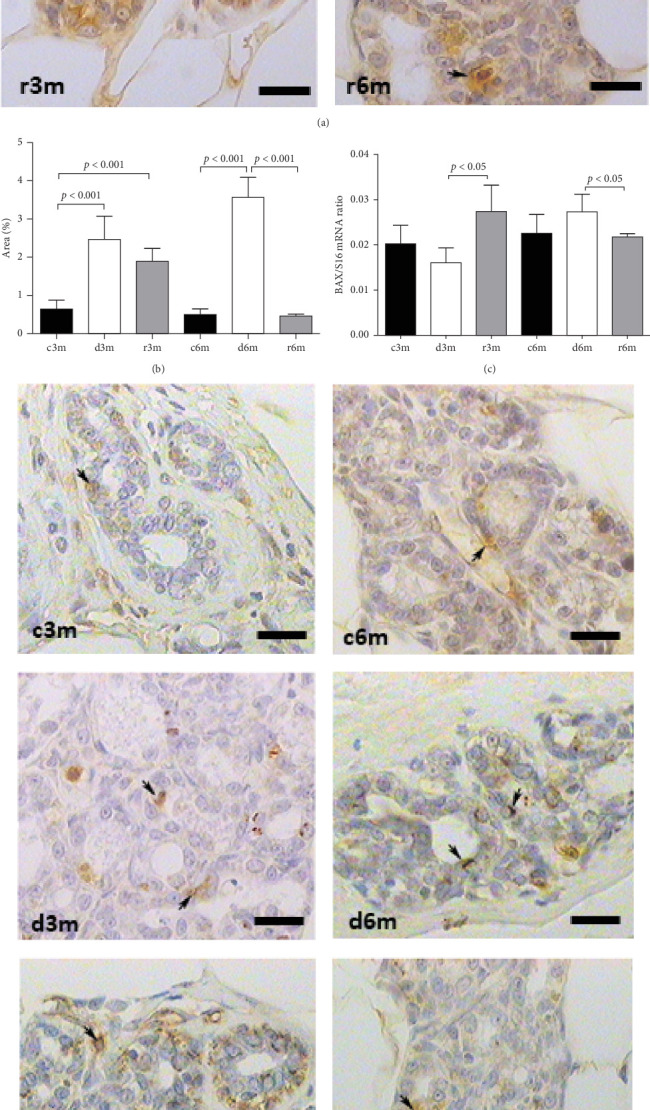
VAD and further refeeding with a VAS diet cause changes in BAX and BCL*2* gene expression in the mammary gland. (A) Anti-BAX immunohistochemistry images of the mammary gland. The black arrows indicate positive immunostaining. magnification 100×. scale bar: 25 µm. (B) Percentage of positive area for anti-BAX immunohistochemistry images shown in (A). (C) Measurement of bax gene expression in the mammary gland as assessed by RT–qPCR. (D) Anti-BCL2 immunohistochemistry images of the mammary gland. magnification 100×. scale bar: 25 µm. (E) Percentage of positive area for anti-BCL2 immunohistochemistry images shown in (A). (F) Measurement of BCL2 gene expression as assessed by RT–qPCR. (G) BAX/BCL2 gene expression ratio from the immunohistochemical images shown in (A) and (D). In (A) and (D), the scale bar is 25 µm, and black arrows indicate positive cells. BAX and BCL2 gene expression levels are shown relative to the expression of the constitutive ribosomal S16 gene. The data shown are representative images or mean values ± SDs (*n* = 4).

Figure 2A shows the results of BAX immunohistochemistry in the mammary glands of the different experimental groups. An increase in BAX immunostaining was observed in the deficient groups at 3 and 6 months for the control and refed groups, respectively. This finding was corroborated by the results of the cellular quantification (Figure 2B). The percentage of BAX ^+^ cells was greater in the vitamin A-deficient groups than in the respective control and refed groups. The observed increases were reversed only after vitamin A supplementation for 1 month in the deprived groups for 5 months. We then corroborated the immunohistochemistry data by measuring BAX gene expression relative to ribosomal S16 gene expression. As shown in Figure 2C, BAX gene expression followed a similar pattern as that observed for the BAX ^+^ cell counts shown in Figure 2B.

Figure 2D shows the results of BCL2 immunohistochemistry in the mammary glands of the different experimental lots, where an increase in BCL2 immunolabeling was observed in the deficient group at 3 months (d3m) compared with the control group (c3m). Refeeding with vitamin A for 3 months did not revert to the values of the controls. In 6-month-old control rats (c6m), little cytoplasmic immunostaining was observed. Compared with rats fed a VAS diet for 6 months, those fed a VAD diet presented increased BCL2 immunostaining for 6 months (d6m vs. c6m). These observations were corroborated by the quantification of BCL2+ (Figure 2E). The number of BCL2+ cells in the mammary gland was greater in the deficient lots than in the respective controls at 3 and 6 months. Figure 2F shows BCL2 gene expression in the mammary glands of the different experimental groups as assessed by RT–qPCR. Figure 2G shows the BAX/BCL2 gene expression ratios as assessed by measuring gene expression by RT–qPCR. In the 3– and 6–month dietary regimens, dietary VAD increased the BAX/BCL2 gene expression ratio in the mammary glands. Refeeding with a VAS diet for 0.5 or 1 month restored the BAX/BCL2 gene expression ratio in the mammary gland. Dietary vitamin A prevents apoptosis in the mammary glands of nulliparous rats.

### 3.5. Dietary Deficiency of Vitamin A Increases Apoptosis in the Mammary Gland Parenchyma

**Figure 3:**
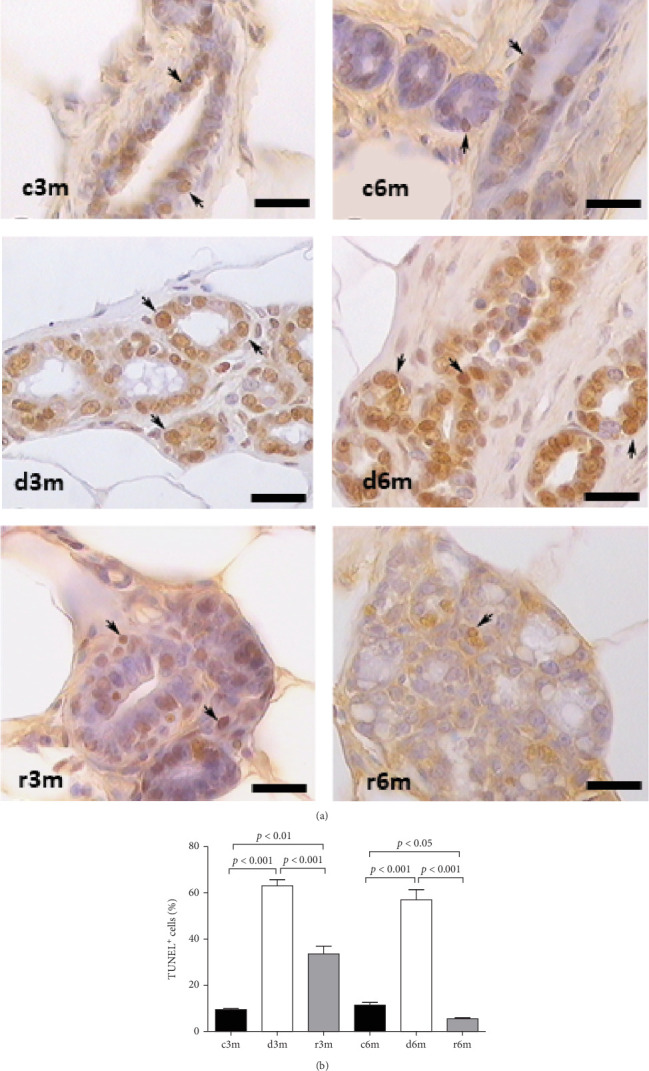
VAD and further refeeding with VAD cause apoptotic changes in the mammary gland. (A) TUNEL immunohistochemistry of mammary glands from the different experimental groups shown in Figure S1. Magnification 100×. The scale bar represents 25 μm, and the black arrow indicates positive nuclei. (B) Percentage of TUNEL-positive cells in the mammary gland. The data are presented as representative images or mean values ± SDs (*n* = 4).

To corroborate the results of the immunohistochemistry and gene expression analysis of molecular markers of apoptosis (Figure 2), we performed a TUNEL assay (Figure 3A). In the mammary glands of the 3-month-old control group, there were few parenchymal cells with apoptotic nuclei. In the d3m group, numerous glandular cells with apoptotic nuclei were observed, indicating a remarkable increase with respect to the control group (d3m vs. c3m). These data are consistent with the decrease in glandular parenchyma observed using H&E staining, as shown in Figure 1. In the 3-month refed group (r3m), the number of apoptotic nuclei was lower than that observed in the deficient group (d3m) but greater than that in the control group (r3m vs. c3m). Moreover, the glandular parenchyma of the r3m group increased considerably compared with that of the deficient group (d3m) (Figure 3A).

The mammary glands of control rats subjected to the 6-month regimen presented very few cells with apoptotic nuclei (c6m). At 6 months, more TUNEL^+^ cells were observed in the deficient group than in the control group (d6m vs. c6m). In the c6m group, normal development of the glandular parenchyma, with secretions inside the acini, was observed. The number of TUNEL^+^ cells in the r6m group was lower than that in the mammary glands of the c6m group (Figure 3A). These data from 3- and 6-month feeding regimens were corroborated by cellular quantification (Figure 3B).

### 3.6. Subchronic Dietary VAD Affects Cell Proliferation in the Mammary Glands of Nulliparous Rats

**Figure 4:**
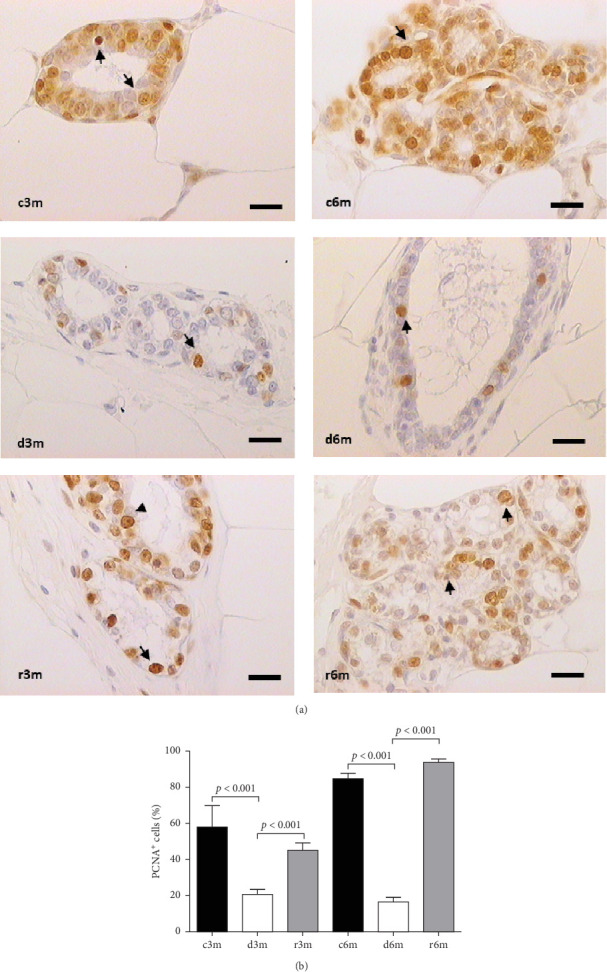
VAD and further refeeding with a VAS diet cause changes in cell proliferation in the mammary gland. (A) Immunohistochemistry for PCNA in the mammary gland. The black arrows indicate positive PCNA + cells. Magnification 100×. The scale bar represents 25 μm. (B) Quantification of the percentage of PCNA^+^ cells from the immunohistochemistry images shown in (A). Data are shown as representative images or mean values ± SDs (*n* = 10).

Cell proliferation is an important tissue response to cell death and tissue dysplasia; thus, we measured PCNA to understand how tissue homeostasis is affected by subchronic dietary VAD.  Figure 4 shows the results of PCNA immunohistochemistry (Figure 4A) and the corresponding quantification of PCNA ^+^ cells (Figure 4B). In the 3-month control group, nuclear immunostaining was observed in the alveolus, whereas in the 3-month deficient group, a decrease in immunostaining was observed in the control group (d3m vs. c3m). In the 3-month refed group, an increase in immunostaining was observed compared with that in the deficient group (r3m vs. d3m). In the 6-month control group, PCNA immunostaining was observed in numerous alveoli and ducts, whereas in the deficient rats in the 6-month group, a decrease in PCNA^+^ cells compared with those in the control group was observed (d6m vs. c6m). Compared with that in the d6m group, there was an increase in PCNA immunostaining in the r6m group. These observations were corroborated by cellular quantification (Figure 4B). Dietary vitamin A maintains secretory cell proliferation in the mammary glands of nulliparous rats.

### 3.7. Dietary VAD Alters the Balance Between Apoptosis and Proliferation

**Figure 5:**
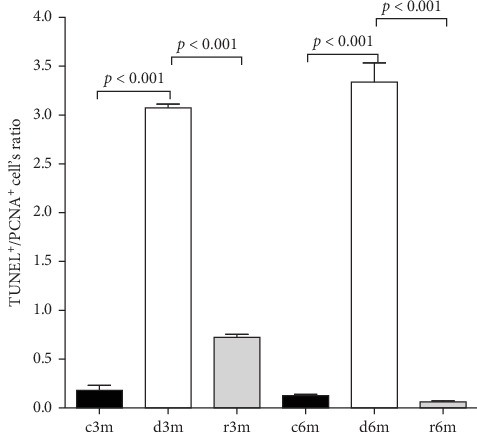
VAS and further refeeding with a VAS diet cause changes in apoptosis and proliferation in the mammary gland. Determination of the TUNEL^+^/PCNA^+^ cell ratios from the immunohistochemistry images shown in Figures 3A and 4A. The data are presented as the means ± SDs (*n* = 4).

The TUNEL^+^/PCNA^+^ cell's ratio increased significantly in the mammary glands of the VAD groups (d3m and d6m) compared with those of the respective controls (c3m and c6m) and refed groups (r3m and r6m) (Figure 5). This antiproliferative but proapoptotic pattern was prevented in the mammary glands of the refeeding groups (r3m and r6m). These data are consistent with a strong modulatory effect of dietary VAD on apoptosis and proliferation in the mammary glands of nulliparous rats.

### 3.8. Subchronic Dietary Deficiency of Vitamin A Inhibits RAR*α* Gene Expression in the Mammary Glands of Nulliparous Rats

**Figure 6:**
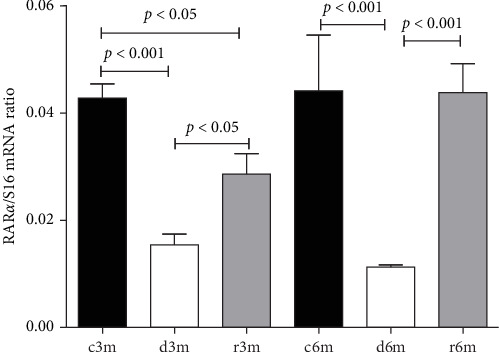
VAD and further refeeding with a VAS diet cause changes in RARα *gene* expression in the mammary gland. RARα gene expression was assessed by RT–qPCR with respect to S16 gene expression in the different experimental groups. The sequences of primers used are shown in Table S4. The data are shown as the mean values of the RARα/S16 ratios ± SDs (*n* = 4).

The expression of RARα in the mammary glands of the rats in the different experimental groups was determined by RT–qPCR (Figure 6). Feeding virgin rats a VAD for 3 or 6 months caused reduced RARα gene expression. Refeeding a VAS diet did not reestablish control levels of RARα gene expression in the 3-month experimental model (C3m vs. r3m). However, 1 month of refeeding in the 6-month experimental model (R6m) reestablished RARα gene expression to that observed in rats fed a VAS diet for 6 months (c6m).

### 3.9. Dietary VAD Causes Inflammation in the Mammary Gland

**Figure 7:**
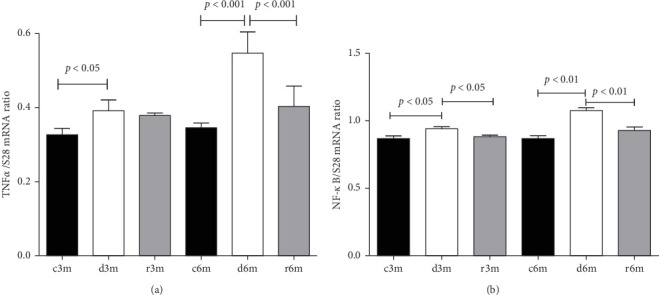
Inflammation response in the mammary glands of rats fed a VAD. (A) TNFα gene expression and (B) NF-κB gene expression. Gene expression was assessed by RT‒qPCR with respect to ribosomal S28 gene expression as a housekeeping gene. The sequences of primers used are shown in Table S4. The data shown are the mean values of the ratios ± SDs (*n* = 8).

Figure 7A shows the TNFα gene expression in the mammary glands of the different experimental groups. At 3 and 6 months of treatment, TNFα expression increased with respect to that in the control groups. We observed a similar expression pattern to that of the TNFα gene when another gene under the transcriptional control of NF-κB (e.g., COX-2) was analyzed (Supporting Information 3: Figure S2). Figure 7B shows NF-κB gene expression in the mammary glands of the different experimental groups. NF-κB gene expression increased in mammary glands exposed to a VAD diet (c3m vs. d3m and c6m vs. d6m). In both experimental models, refeeding with a VAS diet restored NF-κB gene expression to control levels. Dietary VAD causes inflammation in the mammary glands of nulliparous rats.

## 4. Discussion

**Figure 8:**
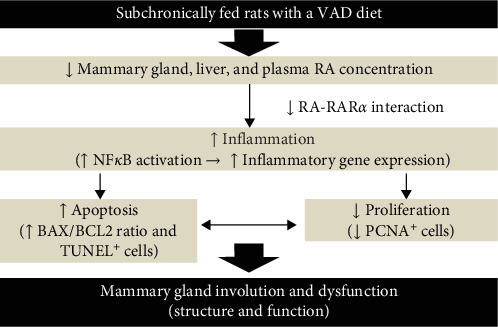
Subchronic dietary vitamin A deficiency causes apoptosis, inflammation, and altered cell proliferation in the mammary glands of nulliparous rats. Schematic representation of the main findings of this study. Subchronic dietary VAD reduces tissue and plasma RA concentrations. This leads to reduced activation of the RA–RARα axis, causing increased activation of the NF-κB inflammatory signaling pathway and the expression of adhesion molecules for inflammatory cells and proinflammatory cytokines. Consequently, the mammary gland parenchyma shows inflammatory cell infiltration and apoptosis but reduced cell proliferation. These changes are consistent with structural and functional alterations that cause mammary gland involution and dysfunction. Mammary gland involution and dysfunction are completely or partially reversed by supplementation with a VAS diet.

To understand the mechanism of mammary gland dysfunction observed in women with dietary deficiency of vitamin A, we used a rat model of subchronic dietary VAD and refeeding a VAS diet (Supporting Information 1: Figure S1). This experimental model of VAD has shown that feeding rats a VAD diet for 3 months results in reduced hepatic and plasma contents of RA and affects the cardiovascular system by altering tissue lipid metabolism and redox status [32–35]. Data from our current experimental model show that dietary vitamin A is critical for the development of the mammary gland in virgin rats. This development is supported by a tightly regulated balance between the anti-inflammatory, antiapoptotic, and pro-proliferative effects of dietary vitamin A to support adequate mammary gland structure and function (Figure 8). Our exciting data are consistent with an inflammation-induced apoptotic process in the mammary glands of nulliparous rats fed a VAD. Inflammation caused by the absence of RA-RARα-mediated inhibition of NF-κB signaling may be, among other factors, a cause for the increased expression of proapoptotic mediators, such as BAX, thereby enhancing apoptosis in the mammary glands of rats fed a VAD.

VAD is common in developing countries without an ongoing food biofortification program [4, 36–38] because vitamin A cannot be synthesized in the body and must be actively obtained from food. This is comprehensively reviewed in [7]. Vitamin A is obtained exclusively through dietary sources, primarily in the form of carotenoids and retinyl esters, which can be delivered to peripheral tissues through chylomicron transport [3]. Liver and adipose tissue constitute the primary sites of vitamin A storage, with ~80%–85% and ~15%–20%, respectively, of total retinyl ester and retinol stores in the body [1, 7]. Bioavailable RA is needed to mediate many physiologically important processes in the body, involving the expression of multiple anti-inflammatory genes and signal transduction pathways [1].

Bartlett et al. [39] reported that the circulating insulin-like growth factor-I concentration decreased in vitamin A–deficient rats. VAD reportedly leads to reduced body weight in a Japanese quail model [40]. This may explain the reduced body weight observed in those rats fed a VAD, and serum retinol concentrations are homeostatically controlled and decrease only when liver stores of vitamin A are very low [41]. The RA concentration within tissues is tightly regulated, with liver and adipose tissue being the main stores in the human body [1]. Vitamin A refeeding led to further body weight regain in the refeeding groups (r3m and r6m).

Plasma concentrations of RA indicate vitamin A status [1, 7]. Retinol levels are reduced during acute inflammation through increased urinary excretion and decreased gastrointestinal absorption [7]. Consistent with our data, the serum and tissue concentrations of RA decreased in rats fed a VAD diet for 3 and 6 months.

The World Health Organization (WHO) recommended high performance liquid chromatography (HPLC) as the method of choice for the determination of retinol in biological samples due to its high sensitivity and specificity [2]. Following this recommendation, the WHO has set a serum retinol concentration of 0.70 μmol/L or below as the cutoff value for the definition of VAD. This threshold is needed to identify populations at risk of deficiency and in need of intervention [2]. Using the rather old method we used in this study [28], we found that feeding rats with a VAD diet for 3 (0.71 ± 0.08 μmol/L) and 6 (0.06 ± 0.03 μmol/L) months results in serum retinol concentrations close to or below this cutoff [2]. These changes are also reflected in the liver and mammary glands (Table 2). The RA concentration we found in the serum of control rats fed a VAS diet (1.79 ± 0.28 μmol/L) is close to the values determined in other studies using HPLC [42, 43].

Under our experimental conditions, the mammary gland concentrations of RA did not reach control levels when the VAS diet was refeeding for 0.5– (r3m) or 1 month (r6m). The greatest impact of VAD was found in the older rats that were exposed to the VAD diet for 6 months. This may be explained by the fact that it is more difficult to reach the homeostatic concentration of RA in older rats when refeeding a VAS diet.

Apoptosis is an important process for controlling the growth of normal and neoplastic breast tissues [44]. The BAX protein can suppress the ability of BCL-2 to block apoptosis [45]. In some tissues, including the breast, stomach, skin, lymph nodes, colon, and small intestine, BAX and BCL2 expression patterns are regulated in parallel, suggesting that there is active antagonism between these two proteins [46]. VAD can cause diseases such as decreased immunity, night blindness, dry skin, diarrhea, and certain cancers, including breast cancer [47]. A recent meta-analysis revealed that in North American and Asian women, high dietary consumption of vitamin A or supplements decreases the incidence of breast and ovarian cancers [48].

Furthermore, RA signaling is needed not only for morphogenesis and development of the gland and adequate milk production but also during the weaning process, when epithelial cell death is coupled with tissue remodeling [49]. These findings are consistent with our findings showing several structural abnormalities in the mammary tissue from our d3m and d6m experimental groups. These abnormalities are reversed by refeeding with a VAS diet.

Transcriptional activation of the nuclear receptor RAR/RXR by RA often leads to inhibition of cell growth [21]. However, in some tissues, RA promotes cell survival and hyperplasia, activities that are unlikely to be mediated by RAR [3]. Opposing effects of RA on cell growth emanate from alternate activation of two different nuclear receptors [50]. Interestingly, our data revealed that refeeding the rats with a VAS diet restored the antiapoptotic/proapoptotic balance in the mammary gland. Furthermore, BAX gene expression was significantly increased and BCL-2 expression was significantly decreased in PC12 cells transduced with Ad-siRAR-α after oxygen‒glucose deprivation-induced injury at the mRNA and protein levels [48]. In agreement with our data, the results of this study suggest that the interaction of RA with its cognate receptor, RARα, inhibits BAX gene expression. Therefore, subchronic deficiency of dietary vitamin A might inhibit this pathway, thus inducing BAX expression and the apoptotic process.

PCNA is a known marker of proliferation that controls mammary gland development. In our 3- and 6-month models of VAD, a significant decrease in PCNA immunostaining was observed compared with that in the control and refed groups. RA positively regulates PCNA expression in embryos [51]. In addition, refeeding with a VAS diet normalizes the cell proliferation marker PCNA and restores mammary gland structure. The *N*-terminal end of PCNA interacts directly with RAREs, thus affecting the transcriptional response to RA in a promoter-specific way [52]. In a VAD model in quails, cell division in the spinal cord, free of RA, decreased in the stages of neural differentiation [53]. Matthews et al. [51] reported a decrease in the cell mass in the pancreas of vitamin A-deficient rats, which was attributed to a reduction in the rate of cell replication.

Our data show that feeding rats a VAD increases the expression of markers of inflammation. RA–RAα exerts an anti-inflammatory effect by affecting the NF-κB and TNFα signaling pathways [54]. For example, a*ll-trans* retinoic acid (RA) inhibits cyclooxygenase-2 and TNFα gene expression induced by bacterial lipopolysaccharide (LPS) in murine peritoneal macrophages [52, 55]. Moreover, RA treatment inhibited IL-12 production in LPS-activated macrophages. This is caused by the competitive recruitment of transcription integrators between NF-κB and RA-RXR for the NF-response elements located in the regulatory region of several proinflammatory and proapoptotic genes [52].

NF-κB and TNFα regulate the apoptosis and involution of the mammary gland. Notably, NF-κB is also involved in inflammatory responses, and it is conceivable that these two signaling pathways govern not only the death/survival balance but also the inflammatory response [48, 55]. The greater TNFα increase in the d6m group than in the d3m group can be explained by the fact that the animals in the 6-month group were older and more prone to inflammatory responses than those in the 3-month group were. Conversely, it is possible that younger animals exposed to a VAD diet respond better than older animals do (d3m vs. r3m and d6m vs. r6m).

One of the main limitations of this study is that although the mammary glands of humans and rats share common features, they also have different structural and developmental traits [56]. For example, apoptosis and inflammation are key processes in both traits, but their regulation and consequences are different, emphasizing the need to consider interspecies differences in mammary gland development and physiology. In particular, these differences determine, among other things, species-specific differences in mammary gland morphogenesis, transcriptome, metabolism, aging and hormonal responses, and mechanisms of cell proliferation, differentiation and death mechanisms [56–58]. Therefore, caution should be taken when interpreting and transferring data, on the effects of VAD in the mammary glands of rats to humans.

In summary, our data are consistent with VAD leading to a reduced negative modulatory effect of RARα-RA competing for NF-κB response elements, which leads to increased inflammation mediators, reduced cell proliferation, and the expression of proapoptotic markers. RA deficiency leads to abnormalities in mammary gland development in nulliparous rats. These effects of VAD can be reversed by vitamin A dietary supplementation.

## Figures and Tables

**Table 1:** Body weight gain in the different experimental groups.

Parameter/group (g)	c3m	d3m	r3m	c6m	d6m	r6m
Initial weight*⁣*^*∗*^	59.50 ± 3.87	55.50 ± 2.88	59.75 ± 2.98	60.75 ± 4.85	54.50 ± 6.45	52.75 ± 5.79
Final weight	251.8 ± 4.86^b^	218.3 ± 6.18^a^	248.0 ± 6.05^b^	341.5 ± 2.88^c^	296.3 ± 16.76^d^	318.5 ± 9.14^e^
Weight gain	192.3 ± 7.17^b^	162.8 ± 3.41^a^	188.3 ± 3.31^b^	280.8 ± 2.22^c^	241.8 ± 1.15^d^	264.5 ± 5.68^c^

(*⁣*^*∗*^) Values are shown as mean values ± SD (*n* = 8). Means with different letters are different (*p* < 0.05).

**Table 2:** Retinoic acid concentrations in serum, liver, and mammary gland.

Serum/tissue	c3m	d3m	r3m	c6m	d6m	r6m
Serum (µmol/L)*⁣*^*∗*^	1.79 ± 0.28^b^	0.71 ± 0.08^a^	1.31 ± 0.08^c^	1.79±0.32^b^	0.06 ± 0.03^d^	1.92 ± 0.27^b^
Liver (µmol/g)	1.56 ± 0.23^b^	0,12 ± 0.06^a^	1.09 ± 0.07^c^	1.71 ± 0.20^b^	0.08 ± 0.03^a^	1.51 ± 0.06^b^
Mammary gland (µmol/g)	0.93 ± 0.09^b^	0.14 ± 0.01^a^	0.25 ± 0.04^a^	0.95 ± 0.04^b^	0.11 ± 0.08^a^	0.36 ± 0.03^c^

(*⁣*^*∗*^) Values are shown as mean values ± SD (*n* = 8). Means with different letters are different (*p* < 0.05).

## Data Availability

The data supporting the findings of this study are available from the corresponding author upon reasonable request. The data that support the findings of this study are openly available in BioRXiv at https://www.biorxiv.org/content/10.1101/2024.06.26.600922v1 [25].

## Supporting Information

Supporting Information 1 Figure S1: Experimental models: (A) Three-month experimental models. (B) Six-month experimental models. The black timeline indicates that the rats were fed a VAS diet. The gray timeline indicates that the rats were fed a VAD. The numbers in the timelines indicate the number of months of diet. c3m, rats fed a VAS diet for 3 months; d3m, rats fed a VAD diet for 3 months; r3m, rats fed a VAD diet for 2.5 months, then fed a VAS diet for 0.5 months; c6m, rats fed a VAS diet for 6 months; d6m, rats fed a VAD diet for 6 months; r6m, rats were fed a VAD diet for 5 months, followed by a VAS diet for 1 month.

Supporting Information 2 Table S1: Preparation of the AIN-93G diet. Table S2: Preparation of the vitamin mixture for the AIN-93G diet. Table S3: Preparation of the mineral mixture for the AIN-93G diet. Table S4: Sequences of primers used in the RT–qPCR measurements.

Supporting Information 3 Figure S2. Cyclooxygenase-2 (COX-2) expression in the mammary glands of rats fed a VAD diet and the effect of refeeding a VAS diet. COX-2 gene expression was assessed via RT–qPCR with respect to ribosomal S28 gene expression as a housekeeping gene. The data shown are the mean values of the ratios ± SDs (*n* = 8). Sequences of primers are shown in Table S4.
