# Supplementary material for: Apoptosis in the Mammary Gland of Virgin Rats Subchronically Fed With a Vitamin A Deficient Diet
Source: Oxid Med Cell Longev. 2025 Jul 14;2025:6334165. doi: 10.1155/omcl/6334165 (PMC12279425; doi:10.1155/omcl/6334165)
Supplement: Supporting Information 2 — Table S1: Preparation of the AIN-93G diet. Table S2: Preparation of the vitamin mixture for the AIN-93G diet. Table S3: Preparation of the mineral mixture for the AIN-93G diet. Table S4: Sequences of primers used in the RT–qPCR measurements. [file 6334165.f2.docx]

**SUPPLEMENTARY TABLES**

**TABLE S1: Preparation of the AIN-93G diet**

| **Components in the AIN-93G diet** | **g/kg diet** |
| --- | --- |
| Casein | 226.0 |
| Dextrin | 132.0 |
| Sucrose | 100.0 |
| Corn oil | 70.0 |
| Fiber | 50.0 |
| Mineral mixture* | 35.0 |
| Vitamin mixture** | 10.0 |
| L-cystine | 3.0 |
| Choline Bitartrate | 2.5 |
| Ascorbic acid | 0.1 |

(*) The composition of the mineral mixture is shown in Table S2,

(**) The composition of the vitamin mixture is shown in Table S3. In the case of the VAD diet the vitamin mixture does not contain *trans*-retinyl palmitate.

**TABLE S2: Preparation of the vitamin mixture for the AIN-93G diet**

| **Components of the mixture** | g/kg mixture |
| --- | --- |
| Nicotinic acid | 3.000 |
| Calcium pantothenate | 1.600 |
| Pyridoxine-HCl | 0.700 |
| Thiamine-HCl | 0.600 |
| Riboflavin | 0.600 |
| Folic acid | 0.200 |
| D-biotin | 0.020 |
| Vitamin B-12 (cyanocobalamin) | 2.500 |
| Vitamin E (500 IU/g) | 15.000 |
| Vitamin A (*trans*-retinylpalmitate)^&^ | 0.800 |
| Vitamin D3 (400,000 IU/g) | 0.250 |
| Vitamin k | 0.075 |
| Sucrose | 974.655 |

(**&**) The vitamin mixture added to the VAS and VAD have the same composition except that in the VAD diet the vitamin mixture does not contain *trans*-retinyl palmitate.

**TABLE S3: Preparation of the mineral mixture for the AIN-93G diet**

| Components in the mineral mixture | mg/kg mixture |
| --- | --- |
| *(a)* Essential mineral elements: | |
| Calcium carbonate, anhydrous | 357.00 |
| Potassium phosphate, monobasic | 196.00 |
| Potassium Citrate, Monohydrate | 70.78 |
| Sodium chloride | 74.00 |
| Potassium sulfate | 46.60 |
| Magnesium oxide | 24.00 |
| Ferric citrate | 6.06 |
| Zinc carbonate | 1.65 |
| Manganese carbonate | 0.63 |
| Cupric carbonate | 0.30 |
| Potassium iodide | 0.01 |
| Sodium selenate, anhydrous | 0.01025 |
| Ammonium paramolybdate.4H_2_O | 0.00795 |
| *(b)* Potentially beneficial elements: | |
| Sodium meta-silicate.9H_2_O | 1.4500 |
| Chromium potassium sulfate.12H_2_O | 0.2750 |
| Lithium chloride | 0.0174 |
| Boric acid | 0.0815 |
| Sodium fluoride | 0.0635 |
| Nickel carbonate | 0.0318 |
| Ammonium vanadate | 0.0066 |
| Sucrose | 221.0260 |

**Table S4: Sequences of primers used in the RT-qPCR measurements**

| Gen | Forward (5'- 3') | Reverse (5'- 3') | Gen Bank Access |
| --- | --- | --- | --- |
| *rarα* | CGCCTGTGAGGGCTGTAAG | ATGCCCACTTCGAAGCATTT | NM_031528 |
| *bcl2* | TGGATGACTGAGTACCTGAAC | AGAGACAGCCAGGAGAAATCAAAC | NM_016993.1 |
| *bax* | TGGTTGCCCTTTTCTACTTTGC | TGATCAGCTCGGGCACTTTA | NM_017059.2 |
| *nfκb* | AGCAACCGAAACAGAGAGG | TTTGCAAAGCCAACCACCAT | NM001276711.1 |
| *tnfα* | GGTGATCGGTCCCAACAAGGA | CACGCTGGCTCAGCCACT | NM012675.3 |
| *cox-2* | CTGTATCCCGCCCTGCTGGTG | ACTTGCGTTGATGGTGGCTGTCTT | U03389.1 |
| *S28* | GTGAAAGCGGGGCCTCACGATCC | GTACTGAGCAGGATTACCATGGC | NR046239.1 |
| *S16* | TCCAAGGGTCCGCTGCAGTC | CGTTCACCTTGATGAGCCCATT | NM_001169146.1 |
